# Evaluation of valve function in antireflux biliary metal stents

**DOI:** 10.1186/s12876-018-0878-8

**Published:** 2018-10-19

**Authors:** Chang-Il Kwon, Jong Pil Moon, Ho Yun, Seok Jeong, Dong Hee Koh, Woo Jung Lee, Kwang Hyun Ko, Dae Hwan Kang

**Affiliations:** 1 Digestive Disease Center, CHA Bundang Medical Center, CHA University, Seongnam, Republic of Korea; 2 Interventional Research Center, M.I.Tech, Co. Ltd., Pyeongtaek, Republic of Korea; 3 Division of Gastroenterology, Department of Internal Medicine, Inha University School of Medicine, Incheon, Republic of Korea; 4 Division of Gastroenterology, Department of Internal Medicine, Hallym University Dongtan Sacred Heart Hospital, Hallym University School of Medicine, Hwaseong, Republic of Korea; 5 Division of Gastroenterology, Department of Internal Medicine, Temple University Hospital, Philadelphia, PA USA; 6 Department of Internal Medicine, Pusan National University School of Medicine and Research Institute for Convergence of Biomedical Science and Technology, Pusan National University Yangsan Hospital, Yangsan, Republic of Korea

**Keywords:** Stent, Biliary stent, Metal stent, Antireflux valve

## Abstract

**Background:**

To overcome duodenobiliary reflux induced by biliary stents, antireflux valve (ARV) biliary stents have been developed and showed improvement in stent patency. However, negative study results have also been reported because stent patency may be decreased by the malfunction of ARV itself. Given such mixed results, the true efficacy of ARV remains unknown and the mechanism of its dysfunction needs to be clearly elucidated. The aim of this study was to investigate the exact mechanism of ARV dysfunction using in vitro phantom models.

**Methods:**

Two experimental models were designed to evaluate two important environmental factors suspected to cause ARV malfunction, i.e. bile flow and pH. Three types of ARV metal stents from different companies were used for the experiments: a funnel type ARV, a windsock type ARV, and a wine glass-shaped ARV. Ten stents of each type were tested (five stents in the bile flow phantom model, and another five stents in the duodenal pH environmental model). To determine ARV malfunction, ARV-induced flow resistance was measured using a custom-made testing device. All stents from the two models were removed every 2 weeks for 12 weeks after stent insertion and were evaluated on morphological and functional changes of the ARV.

**Results:**

Only ARV of wine glass-shaped ARV was morphologically changed due to silicone bond detachment in the bile flow model. All types of ARV were morphologically changed in the pH model. The morphological changes of ARV influenced the flow resistance. The antegrade pressure gradients were increased over time in the pH model (*p* < 0.05).

**Conclusions:**

Morphological change of the ARVs may induce dysfunction of ARV metal stents, which is mainly due to duodenal pH environment. In the future, development of new ARV that is not affected by duodenal environmental factors can be expected to improve stent patency.

**Electronic supplementary material:**

The online version of this article (10.1186/s12876-018-0878-8) contains supplementary material, which is available to authorized users.

## Background

One of the most important milestones in therapeutic endoscopy was the development of biliary stent, which allowed a non-surgical approach to resolve obstructive jaundice, and preoperative stabilization of patients [1]. Over the last 30 years, biliary stents have surpassed their fundamental indication, which is palliative treatment of malignant biliary obstruction, and expanded their scope to include various benign biliary diseases.

The basic plastic stents are cheap and effective, but have short stent patency due to their limited diameter that can be deployed through the working channel of the endoscope [2]. To overcome this shortcoming, self-expandable metal stents (SEMS) were developed with wide luminal diameters, and thus longer stent patency. Therefore, SEMS have been most widely used for palliation of malignant biliary obstruction and allow patients to avoid repetitive procedures, which is helpful for reducing medical costs and improving their quality of life [3–5]. However, the wide luminal diameter of SEMS has its own limitations. The larger orifice of duodenal papilla easily induces duodenobiliary reflux, leading to ascending infection or early stent malfunction due to food hanging or impaction [6, 7].

As a way of ensuring a longer stent patency, the plastic stents with an antireflux valve (ARV) which prevents duodenobiliary reflux was developed and showed positive result [8]. After that, various types of ARVs have been applied to SEMS and most of them also produced positive results, raising expectations to improve the function of stents drastically [9–13]. However, negative study results have been also reported because stent patency may be decreased by the malfunction of ARV itself [14]. The mechanism of ARV malfunction was speculated in the early days that ARV was pressed against the duodenal wall because of migration, malposition, or bowel motion, or ARV became harder with time due to direct cancer invasion or gastroduodenal secretion [15–17]. In addition, as opposed to the initial report, a recent prospective randomized study demonstrated high rate of occlusion of ARV plastic stents due to ARV dysfunction [18]. Given such mixed results, the true efficacy of ARV SEMS remains unknown and the mechanism of its dysfunction needs to be clearly elucidated.

We intended to experimentally investigate whether ARV SEMS malfunction is universal and if so, which environmental factors affect the ARV SEMS function the most, and which factors were ignored or not clarified in previous studies. The aim of this study was to evaluate the efficacy of biliary ARV SEMS and the mechanism of ARV dysfunction using in vitro phantom models.

## Methods

This was a pre-clinical, in vitro phantom experiment, a proof-of-concept study. The experimental models were designed to evaluate two important environmental factors, bile flow and pH, which are suspected to cause ARV malfunction.

### Antireflux valve SEMS

**Fig. 1:**
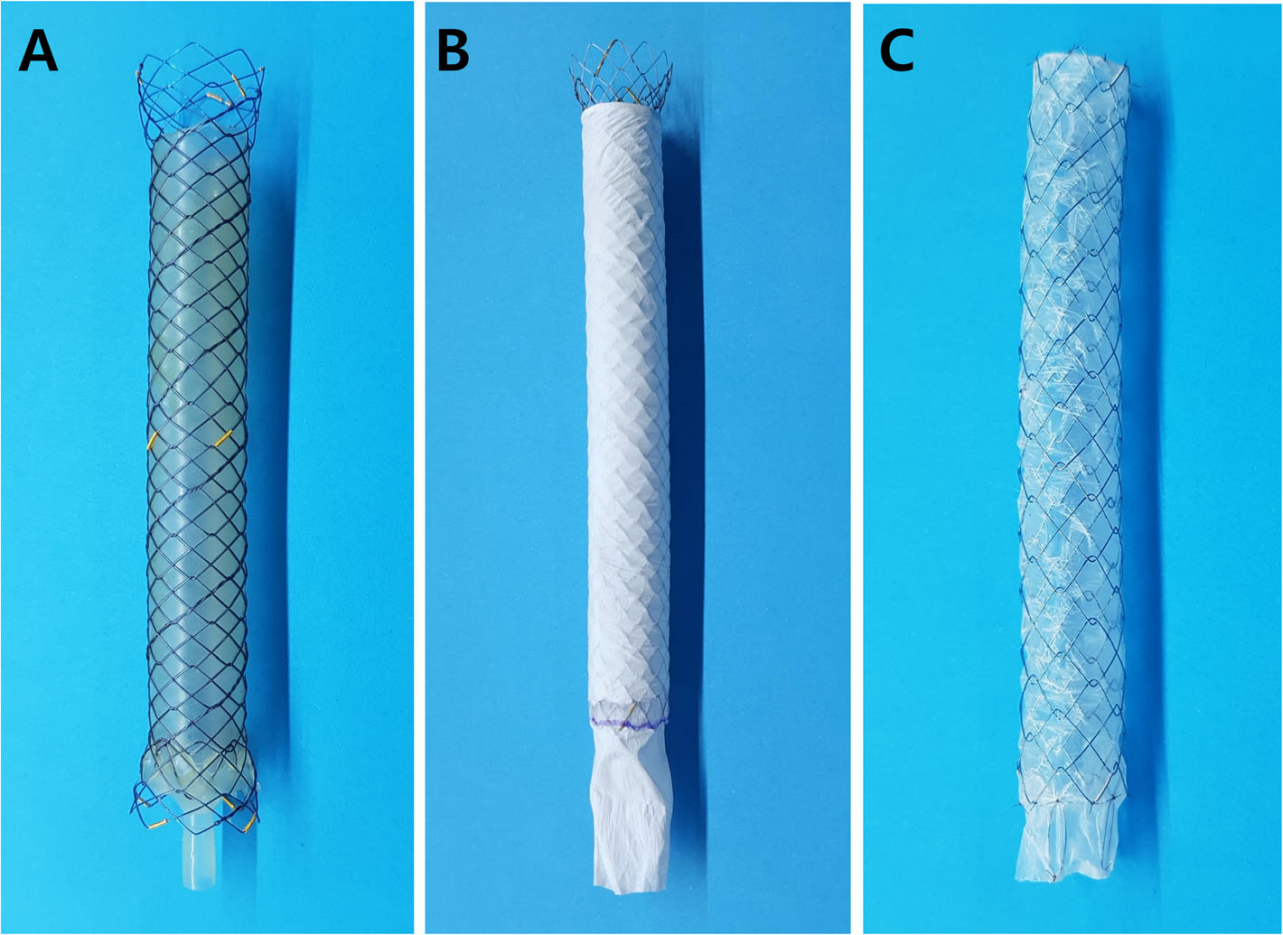
Three types of metal stents with antireflux valve. **a** A wine glass-shaped antireflux valve stent. **b** A windsock type antireflux valve stent. **c** A funnel type antireflux valve stent

Out of the ARV SEMS of which the results have been reported previously, the following three types of ARV SEMS from different companies were used for the experiments: a wine glass-shaped ARV (Hanarostent; M.I.Tech, Co. Ltd., Pyeongtaek, Korea), a windsock type ARV (EGIS Biliary Stent M-Valve; S&G Biotech, Seongnam, Korea), and a funnel type ARV (Niti-S ComVi; Taewoong Medical Inc., Gimpo, Korea) (Fig. 1) [11, 13, 14]. Five stents of each type were tested in the bile flow phantom model, and another five stents of each type in the duodenal pH model.

Radial force was measured at the center of the SEMS using the Push-Pull Gauge (DS2-50 N, IMADA Inc., Japan), and the mean value was calculated by measuring five SEMSs for each type: a wine glass-shaped ARV SEMS, 340.0 gf; a windsock type ARV SEMS, 105.7 gf; and a funnel type ARV SEMS, 245.7 gf (Additional file 1: Figure S1). Axial force was measured using the UTM (Lloyd Instruments LRX PLUS, AMETEK Inc., PA, USA). With exception of the distal end of SEMS with ARV about 40 mm, all SEMSs were fixed to the frame. Axial force was measured by bending the unfixed area, and the mean value was calculated by measuring five SEMSs for each type: a wine glass-shaped ARV SEMS, 0.720 N; a windsock type ARV SEMS, 0.181 N; and a funnel type ARV SEMS, 0.155 N (Additional file 2: Figure S2).

### In vitro bile flow phantom model

**Fig. 2:**
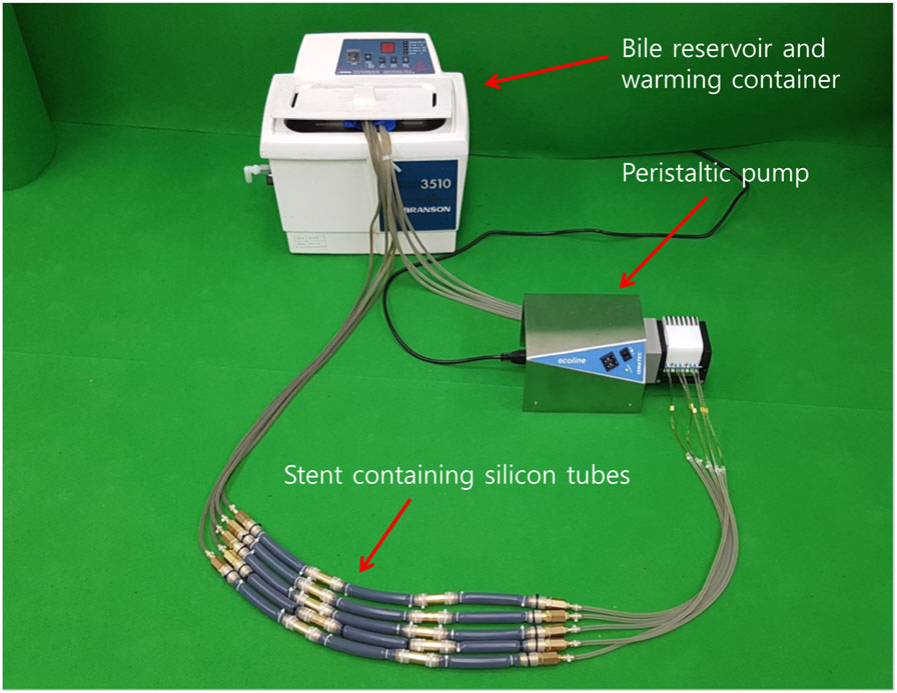
An experimental bile perfusion system for the in vitro bile flow phantom model. This system consists of a peristaltic pump, a bile reservoir, a bile reservoir warming container, five stent containing silicone tubes, and ten connecting silicone tubes. Three types of antireflux metal stents were inserted into the silicon tubes (15 stents in total)

To determine whether ARV malfunction could be caused by bile, presumably the most important factor affecting ARV, an experimental bile perfusion system was made based on previous study reports (Fig. 2) [19–21]. This system was comprised of a peristaltic pump (ECOLINE, ISM 1077A; ISMATEC, Wertheim, Germany) to provide the power to circulate bile, a bile reservoir to contain circulating bile, a bile reservoir warming container (BRANSONIC, 3510E-DTH; Banson Ultrasonics, Danbury, CT) to maintain bile temperature at the same level as body temperature, and silicone tubes which housed 15 ARV SEMS. Human bile obtained after the recovery of cholangitis in a patient with percutaneous transhepatic biliary drainage or percutaneous transhepatic gallbladder drainage was replaced every 3–4 days, 600 mL each time. The peristaltic pump speed was set to the lowest value at 1 mL/min. All stents were removed from the device to measure the flow resistance every 2 weeks for 12 weeks - a total of 6 times - and then placed back to the silicone tubes with its position switched each time.

### In vitro duodenal pH environmental model

**Fig. 3:**
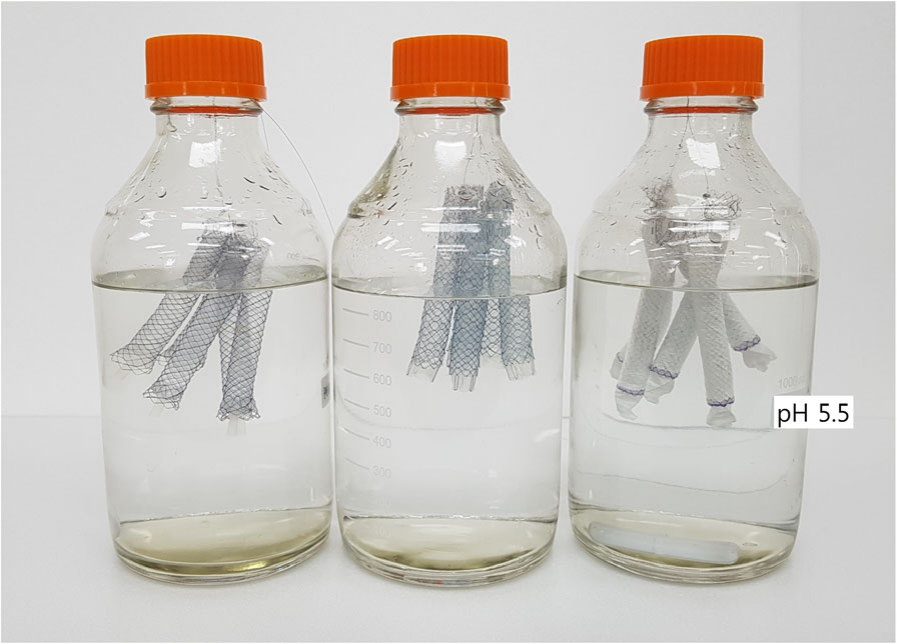
An experimental duodenal pH environmental model. Distilled water at pH 5.5 was put in the reservoirs, and 5 antireflux stents of each type were placed so that the distal part could be submerged

This model was designed to determine the effect of duodenal pH on the function of ARV SEMS. Unlike the flow phantom model, this model was made into a static state to simulate the duodenal environment. PBS solution (phosphate buffered saline in sterile distilled water) and pH 2.0 buffer solution were mixed and put in the fluid reservoirs. Then the pH tester (SX620, SANXIN, Sanghai, China) was used to maintain at pH 5.5, with the pH checked every 3–4 days to constantly adjust the pH. Similar to the bile flow phantom model, a total of 15 ARV SEMS were used, with the ARV submerged in the fluid (Fig. 3). All stents were again removed from the model to measure the flow resistance every 2 weeks for 12 weeks - 6 times in total.

### Evaluation of flow resistance

To determine ARV malfunction, ARV-induced flow resistance was measured. All 30 stents from the two models were removed every 2 weeks for 12 weeks after the insertion to measure the degree of pressure gradient using a custom-made testing device with glycerin fluid. Glycerin fluid (glycerin in sterile distilled water, 60 cP ± 5%) has a similar viscosity as human bile, and the viscosity was continuously adjusted using the Viscometer (BROOKFIELD LVDV-II + Pro, Brookfield Engineering, Middleboro, MA). Setting and conditions were as follows; 62 LV Spindle, RPM 60, Torque 10~ 100%, ±23 °C.

**Fig. 4:**
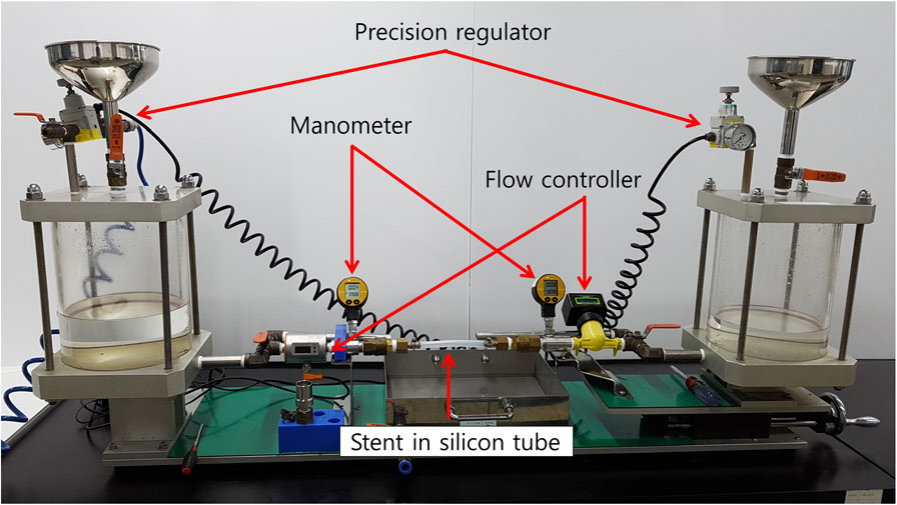
The flow resistance system. The device was custom-made and consists of a frame, precision regulators, manometers, flow controllers, and two water tanks. Glycerin fluid was put in both water tanks

The flow resistance measuring system was produced by M.I.Tech as reported in the previous studies (Fig. 4) [8, 14]. The system consists of a frame (BJ-Tech, Seoul, Korea), precision regulators (RP1000-8-02; CKD Corporation, Komaki, Aichi, Japan), manometers (Leo 2, Mano 2000; Keller, Winterthur, Switzerland), flow controllers (SM60000; IFM Electronic, Essen, Germany), and two water tanks. Glycerin fluid was put in both water tanks. The precision regulators maintained a constant pressure of fluid influx into the tank.

An ARV SEMS was fixed in the silicon tube. To determine the antegrade flow resistance, the glycerin fluid was made to flow through the ARV SEMS at 0.25, 0.5, 0.75, 1.0 and 1.25 L/min using the flow controller. The maximum pressure read on the manometer was considered as the flow rate of the solution. The flow rate of each ARV SEMS was measured three times and the mean value was considered the pressure at each flow rate. Before each measurement of flow resistance, the flow resistance of the silicon tube itself without stent was measured at each flow rate and the average value was used to correct the stent flow resistance.

### Statistical analysis

The mean, standard deviation (SD), and range were used to summarize the data for continuous variables. Wilcoxon signed rank test was used to analyze the changes of the flow resistance. *P <* 0.05 was considered statistically significant. Statistical analysis was performed with IBM® SPSS® Statistics (Version 21.0.0; SPSS Inc., Chicago, IL, USA).

## Results

### Flow resistance in the bile flow phantom model

**Fig. 5:**
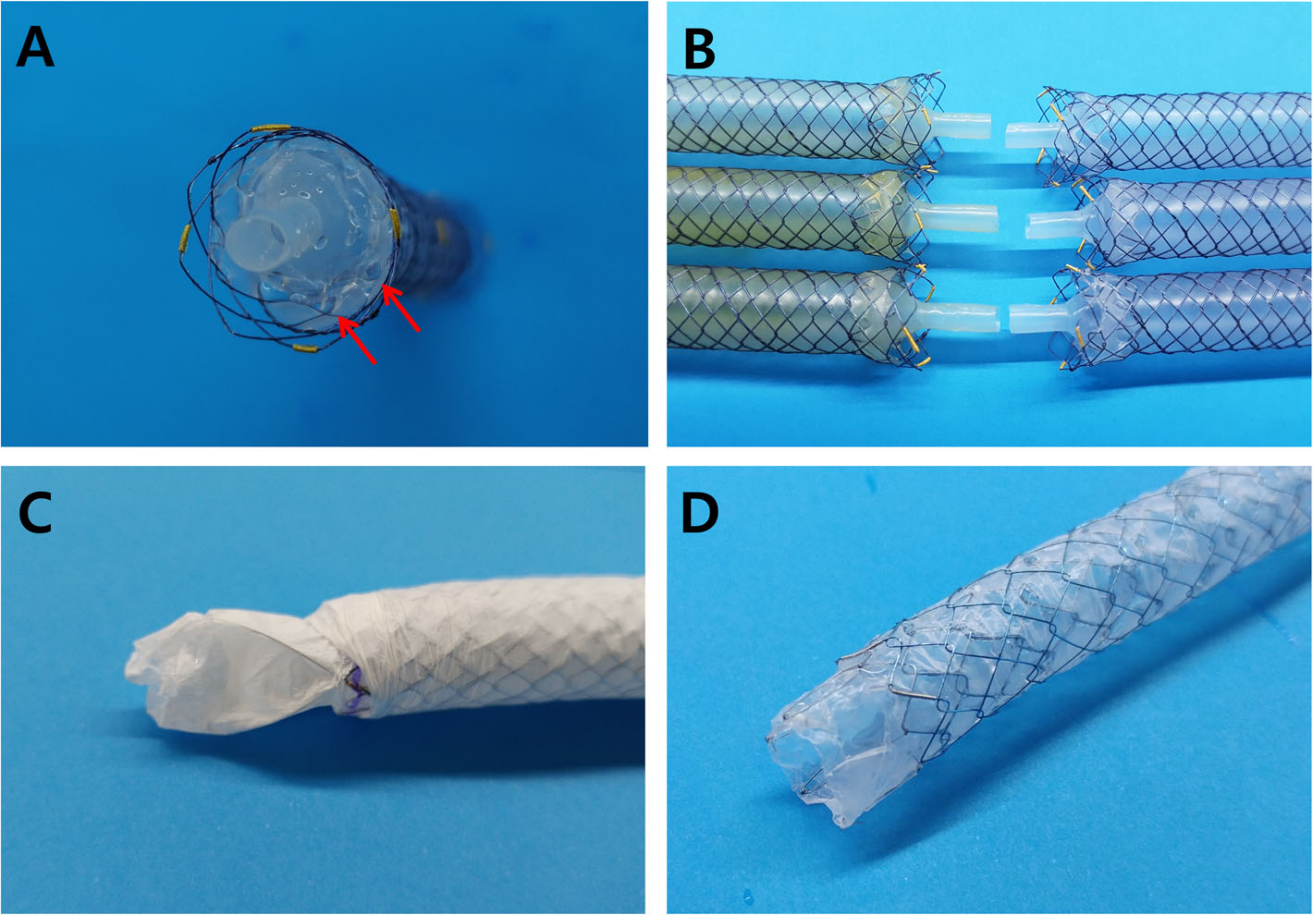
Morphologic changes of antireflux. **a** The wine glass-shaped antireflux valve is deformed, creating small holes (arrow) in the part connected to the stent body. **b** Compared to the wine glass-shaped antireflux valves used in the bile flow phantom model on the left, the antireflux valves used in the duodenal pH model on the right became relatively thicker, shorter, or deformed. **c** Windsock-type antireflux valve shape deformed due to the skirt clinging to itself. **d** *funnel* type antireflux valve shape deformed due to the skirt clinging to itself

**Fig. 6:**
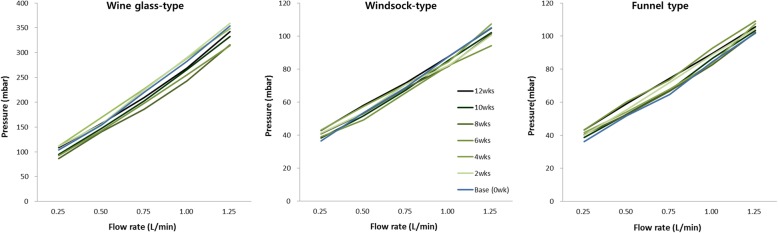
Flow chart of flow resistance in the bile flow phantom model. In all 3 types (a wine glass type, a windsock type, and a funnel type), the mean flow resistance changes randomly rather than increasing consistently in 2 week interval

The valve of wine glass-shaped ARV became detached from one stent at Week 6 and another stent at Week 10, and thus they were excluded from the flow resistance evaluation (Fig. 5a). With only 3 stents left with the wine glass-shaped ARV, the comparison for statistical significance was not feasible due to the small sample size. However, the flow resistance did not increase significantly at each flow rate. On the contrary, the flow resistance showed a statistically significant increase with the windsock type at a low flow rate and the funnel type at every flow rate (Table 1). However, as shown in Fig. 6, the mean flow resistance changed randomly rather than increasing consistently in 2 week interval. This suggests there likely was another mechanism affecting the flow resistance rather than true ARV dysfunction. Therefore, even though the increase in flow resistance between initial value and final value was statistically significant, this result was interpreted as not representative of ARV dysfunction.

**Table 1:** Changes of flow resistance in the bile flow phantom model

Type of antireflux valve	Flow rate (L/min)	Flow resistance (mbar, mean ± standard deviation)		*p*-value
		Baseline	12 weeks	
Wine glass type (*n* = 3)	0.25	103.8 ± 2.9	105.8 ± 2.3	0.109
	0.5	154.0 ± 4.5	166.4 ± 9.5	0.109
	0.75	219.8 ± 5.3	230.8 ± 17.3	0.180
	1.0	282.6 ± 10.3	305.5 ± 23.4	0.109
	1.25	353.6 ± 8.6	400.0 ± 44.3	0.285
Windsock type (*n* = 5)	0.25	36.4 ± 0.9	42.8 ± 0.9	0.039
	0.5	53.6 ± 1.9	58.0 ± 2.9	0.042
	0.75	69.0 ± 1.7	71.6 ± 2.8	0.223
	1.0	87.6 ± 0.5	87.6 ± 1.7	1.000
	1.25	105.0 ± 0.7	104.8 ± 1.6	0.854
Funnel type (*n* = 5)	0.25	36.2 ± 1.3	43.2 ± 2.0	0.039
	0.5	51.8 ± 0.8	59.2 ± 2.1	0.042
	0.75	64.6 ± 1.1	74.4 ± 2.9	0.042
	1.0	84.4 ± 1.8	89.4 ± 2.9	0.078
	1.25	101.8 ± 1.1	105.6 ± 1.7	0.042

Data were analyzed by using the Wilcoxon signed rank test

### Flow resistance in the duodenal pH model

**Fig. 7:**
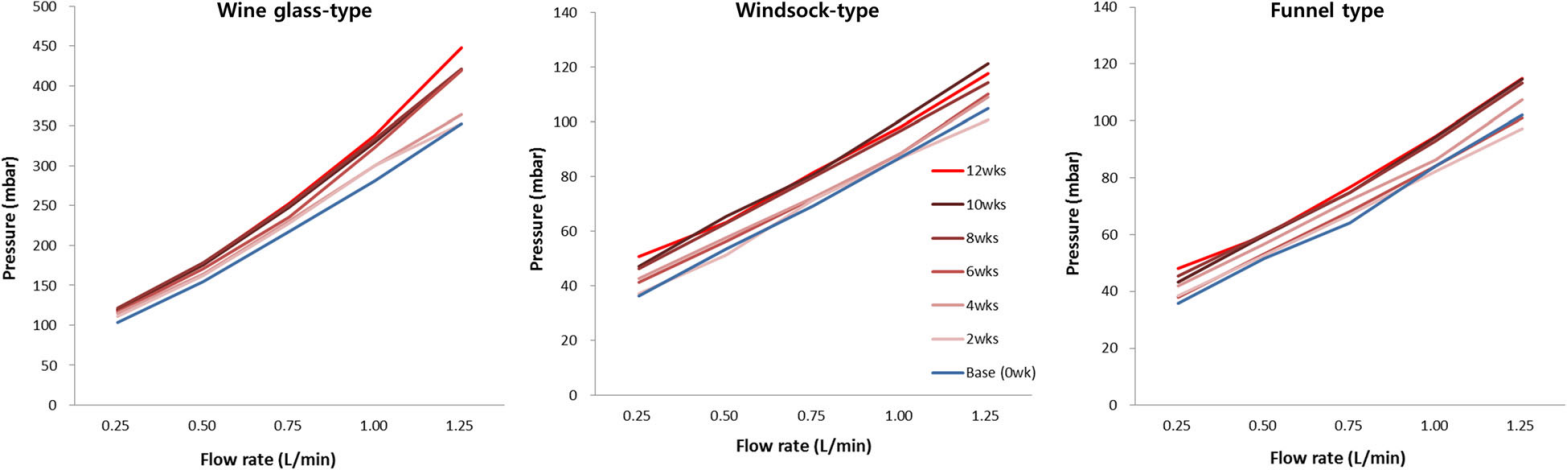
Flow chart of flow resistance in the duodenal pH model. In all 3 types (a wine glass type, a windsock type, and a funnel type), the mean flow resistance appears increasing constantly every 2 weeks interval

As have occurred in the bile flow phantom model, two of wine glass-shaped ARV became detached from its stent at Week 2 and 6, thus they were excluded from the flow resistance evaluation. Again, with only 3 stents left with the wine glass-shaped ARV, the comparison for statistical significance was not feasible due to the small sample size. The flow resistance was noted to increase at every flow rate. Both the windsock type and the funnel type had a statistically significant increase at every flow rate (Table 2). In addition, as shown in Fig. 7, there was constant increase in flow resistance every 2 weeks. Unlike the bile phantom model, this was considered to be associated with the pH-induced morphologic changes (hardening, distorting or sticking) of the ARVs over time (Fig. 5b-d).

**Table 2:** Changes of flow resistance in the duodenal pH model

Type of antireflux valve	Flow rate (L/min)	Flow resistance (mbar, mean ± standard deviation)		*p*-value
		Baseline	12 weeks	
Wine glass type (*n* = 3)	0.25	103.2 ± 2.4	118.9 ± 2.4	0.109
	0.5	154.6 ± 4.7	177.5 ± 10.0	0.109
	0.75	217.8 ± 4.1	253.0 ± 17.3	0.109
	1.0	281.6 ± 8.9	338.5 ± 23.5	0.109
	1.25	352.6 ± 7.9	447.4 ± 44.4	0.109
Windsock type (*n* = 5)	0.25	36.2 ± 0.8	50.8 ± 2.6	0.043
	0.5	53.4 ± 1.8	63.3 ± 1.9	0.043
	0.75	69.4 ± 1.6	81.5 ± 1.8	0.041
	1.0	87.2 ± 0.8	98.1 ± 4.0	0.043
	1.25	104.8 ± 0.8	117.2 ± 5.3	0.043
Funnel type (*n* = 5)	0.25	35.8 ± 0.8	48.0 ± 2.0	0.043
	0.5	51.6 ± 0.5	59.7 ± 2.7	0.043
	0.75	64.2 ± 0.8	76.7 ± 1.5	0.039
	1.0	84.2 ± 1.6	94.7 ± 2.2	0.041
	1.25	102.0 ± 0.7	114.9 ± 4.0	0.043

Data were analyzed by using the Wilcoxon signed rank test

As a note, the measured retrograde flow rates by the ARV were so high that they deviated from the measurement range even at a very slow flow rate, as previously reported [8, 14]. Unable to make out the differences between the groups, retrograde flow resistance was excluded in the results.

## Discussion

To best of our knowledge, this was the first experimental study which demonstrated the malfunction of ARV of SEMS caused by the morphologic changes of the ARV. This study also revealed that the possible mechanism of the malfunction was due to the duodenal pH rather than the bile flow.

To facilitate the understanding of these experiments, let us first describe how to make the ARVs. There are two types of ARVs (Table 3). The first one is the skirt type used for covered SEMS where the covered sheath is made longer (windsock type and funnel type) [11, 13]. This type uses polytetrafluoroethylene (PTFE) membrane for the covered sheath so that the ARV can sway freely like a skirt, and is made as an integral part of the body. It can maintain a relatively large diameter for the ARV, and without the need for thicker diameter of SEMS and larger stent introducer, it can be easily inserted in the working channel of the duodenoscope. However, it is known to form biofilms more easily than silicone membrane, more difficult to position in the biliary tract for longer ARVs, and the valve may become deformed over time depending on the ARV length [18, 22]. The second type is produced by attaching an ARV (a wine glass type, S-shaped type, and a nipple type) to existing SEMS (uncovered- or covered-SEMS) with silicon glue [9, 12, 14]. This method uses silicone membrane to attach the ARV to the body and, due to the nature of silicone, may not sway like the skirt type but easily clings to itself. For this reason, it needs to be in a relatively thick shape and uses a silicone bond to let it attach to the body. Without the long skirt, it can be positioned easily in the biliary tract and does not become deformed by the surrounding structure. The major shortcoming is that the ARV shape makes the inner diameter much smaller than the original inner diameter of the metal stent, which greatly affects the bile flow. In addition, the thickness of the ARV itself requires a larger outer diameter, which makes it difficult to insert in the working channel of the duodenoscope, and the ARV can become detached because the integrity of the silicone glue may be compromised by the surrounding environment, as seen from the wine glass type ARV used in our experiment. These flaws of both types of ARV seem to suggest that malfunction of the ARV may be significant.

**Table 3:** Classification of antireflux metal stent

Creating method	Valve shape	Valve material	References	Advantages	Disadvantages
Skirt type	Windsock type	e-polytetrafluoroethylene	Lee YN, et al. [13]	Wide inner diameter Thinner introducer	More biofilm formation Difficult positioning Possible valve deformity
	Funnel type	polytetrafluoroethylene	Hamada T, et al. [11]		
Attachment type	Nipple type	Silicone	Hu B, et al. [9]	Easy positioning Better maintenance of valve shape	Narrow inner diameter Thick membrane Thicker introducer Bonding and detachment
	S-shaped type	Silicone	Lee KJ, et al. [12]		
	Wine glass type	Silicone	Kim DU, et al. [14]		

Considering these strengths and weaknesses, it is expected that there will be main limitations in the manufacturing of ARVs to make functional metal stents with a better outcome than the existing SEMS. However, most of the articles on ARV SEMS published so far indicate favorable results. Recently, the possibility of ARV malfunction was objectively presented in the prospective clinical study of ARV plastic stent showing unfavorable results as opposed to the initial favorable report [8, 18]. Plant material in duodenal contents is suspected as the cause of ARV plastic stent malfunction as it is one of important factors causing the plastic stent malfunction, but other unknown factors are speculated to produce a synergistic effect [17, 23]. Likewise, we suspected that ARV SEMS may have ARV malfunction due to a synergistic effect from various unknown factors. Thus, we planned and executed these experiments to investigate this possibility and its mechanism.

The effect of bile on the wine glass-shaped ARV was inconclusive, as two ARVs were detached from the SEMS, rendering statistical analysis not feasible. However, this may represent an important mechanism of dysfunction for the wine glass-shaped ARV, as another two ARVs in pH group became detached as well. The effects of bile on windsock and funnel type of ARVs were also inconclusive. Although the increase in flow resistance for these stents was statistically significant, this increase did not occur consistently in 2 week interval (Table 1 and Fig. 6). This can be explained by the formation of biofilm or sludge (Fig. 5b). The biofilm or sludge are easily cleared during each measurement, which would ‘reset’ or decrease the flow resistance when placed back into the silicone tube in the bile flow phantom model. This resulted in flow resistance level which did not increase with every 2 week measurements. Because the increase in the flow resistance was not consistent for windsock and funnel ARVs, the flow of bile could not be concluded as a cause of stent dysfunction. Also, the weakly alkaline bile does not induce deformities of the ARV shape.

The flow resistance in the duodenal pH model revealed the following facts (Table 2 and Fig. 7): firstly, the wine glass-shaped ARV, attached with an adhesive, may become detached due to the weakly acidic duodenal pH, which also makes the ARV deformed, shorter, and thicker. Secondly, the duodenal pH also deforms the polytetrafluoroethylene membrane, which is also shown by the gradual change of the mean flow resistance over time. The actual clinical examples of ARV shape becoming markedly shorter and thicker are presented in the previous report [14].

Taken together, the ARV may be affected by the surrounding environment of the inserted stent (bile, duodenal pH, etc.) over time, mostly morphologic changes leading to a malfunction. It is speculated that the effect from the surrounding environment was greater with the duodenal pH than with the bile. To overcome this issue, attempts can be made to deploy ARV metal stents fully into the biliary tract or to develop ARVs in shapes and materials that are resistant to morphologic changes and can maintain their function for as long as possible to achieve a better functionality. In this context, the pH-induced morphologic changes are worth considering for ARV esophageal metal stents as well. Various types of ARV esophageal metal stents were introduced on the market with favorable clinical outcomes but not being used widely now, and it can be cautiously speculated that the same issues are involved [24–26]. Because ARV malfunction is highly likely with gastric pH, which is more acidic than duodenal pH, we hope this will be investigated further in the future.

The limitations of this proof-of-concept study are as follows: (1) This study used in vitro models, instead of in vivo animal model or human study, and results from in vivo or human study can be substantially different from this proof-of-concept study. This is even more significant considering the environment of duodenal lumen, which would be considerably different from biliary tract and may result in faster or more severe dysfunction of ARVs; (2) a small number of stents was used in this phantom model; and (3) only two factors (bile flow and duodenal pH) were tested, among the environmental factors that might affect the stent malfunction; (4) In order to solve the ‘reset’ problem of cleaning the biofilm or sludge during flow measurement in the bile flow phantom model, more sets should be mounted to remove one set every 2 weeks and measured separately. That way we could get a more accurate measurement. However, this would have resulted in prohibitively high material cost, and the silicone tube cannot be made very long while the bile flow is maintained. Despite these limitations, our proof-of-concept study successfully analyzed and compared stent flow rate by different ARV shape. Dysfunction of ARV SEMS has been only speculated, but not demonstrated so far. Our results may be helpful in providing basic data for further development of functional metal stents, and selecting an ARV metal stent in the clinical settings.

## Conclusions

Morphological change of the ARVs may induce dysfunction of ARV metal stents, which is mainly due to duodenal pH environment. In the future, development of new ARV that is not affected by duodenal environmental factors can be expected to improve stent patency.

## Additional files

Additional file 1: **Figure S1.** Radial force measurement setup. Radial force was measured at the center of the SEMS using the Push-Pull Gauge (DS2-50 N, IMADA Inc., Japan). A and B. A wine glass-shaped ARV SEMS. C. A windsock type ARV SEMS. D. A funnel type ARV SEMS. (TIF 3990 kb)

Additional file 2: **Figure S2.** Axial force measurement setup. Axial force was measured using the UTM (Lloyd Instruments LRX PLUS, AMETEK Inc., PA, USA). With exception of the distal end of SEMS with ARV about 40 mm, all SEMSs were fixed to the frame. Axial force was measured by bending the unfixed area. A and B. A wine glass-shaped ARV SEMS. C. A windsock type ARV SEMS. D. A funnel type ARV SEMS. (TIF 4454 kb)

## Abbreviation

ARV: Antireflux valve
PTFE: Polytetrafluoroethylene
SD: Standard deviation
SEMS: Self-expandable metal stents
