# Supplementary figures and images for: Evaluation of valve function in antireflux biliary metal stents
Source: BMC Gastroenterol. 2018 Oct 19;18:150. doi: 10.1186/s12876-018-0878-8 (PMC6194677; doi:10.1186/s12876-018-0878-8)

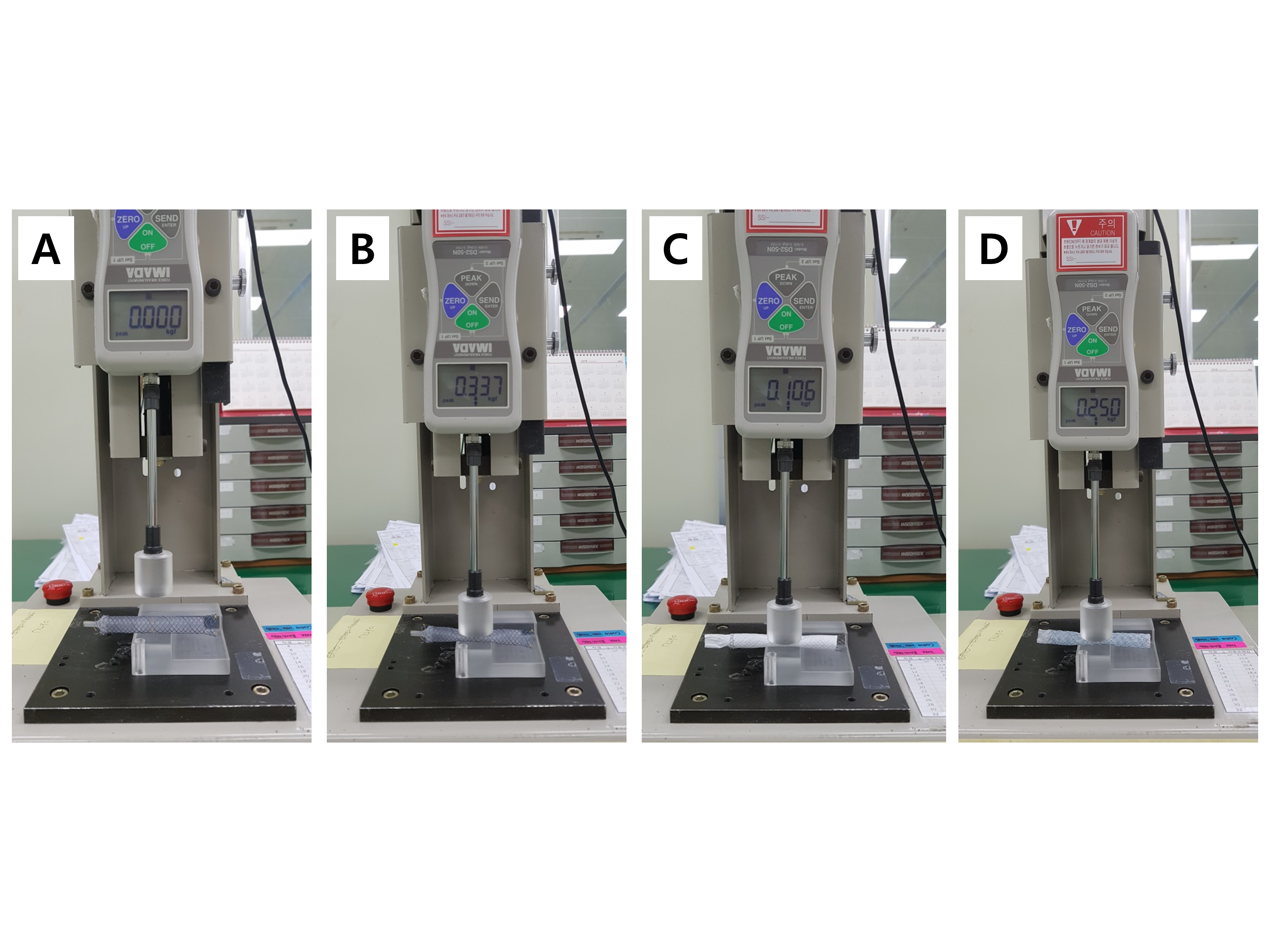

Supplement: Supplementary file 1 — Figure S1. Radial force measurement setup. Radial force was measured at the center of the SEMS using the Push-Pull Gauge (DS2-50 N, IMADA Inc., Japan). A and B. A wine glass-shaped ARV SEMS. C. A windsock type ARV SEMS. D. A funnel type ARV SEMS. (TIF 3990 kb) [file 12876_2018_878_MOESM1_ESM.tif]

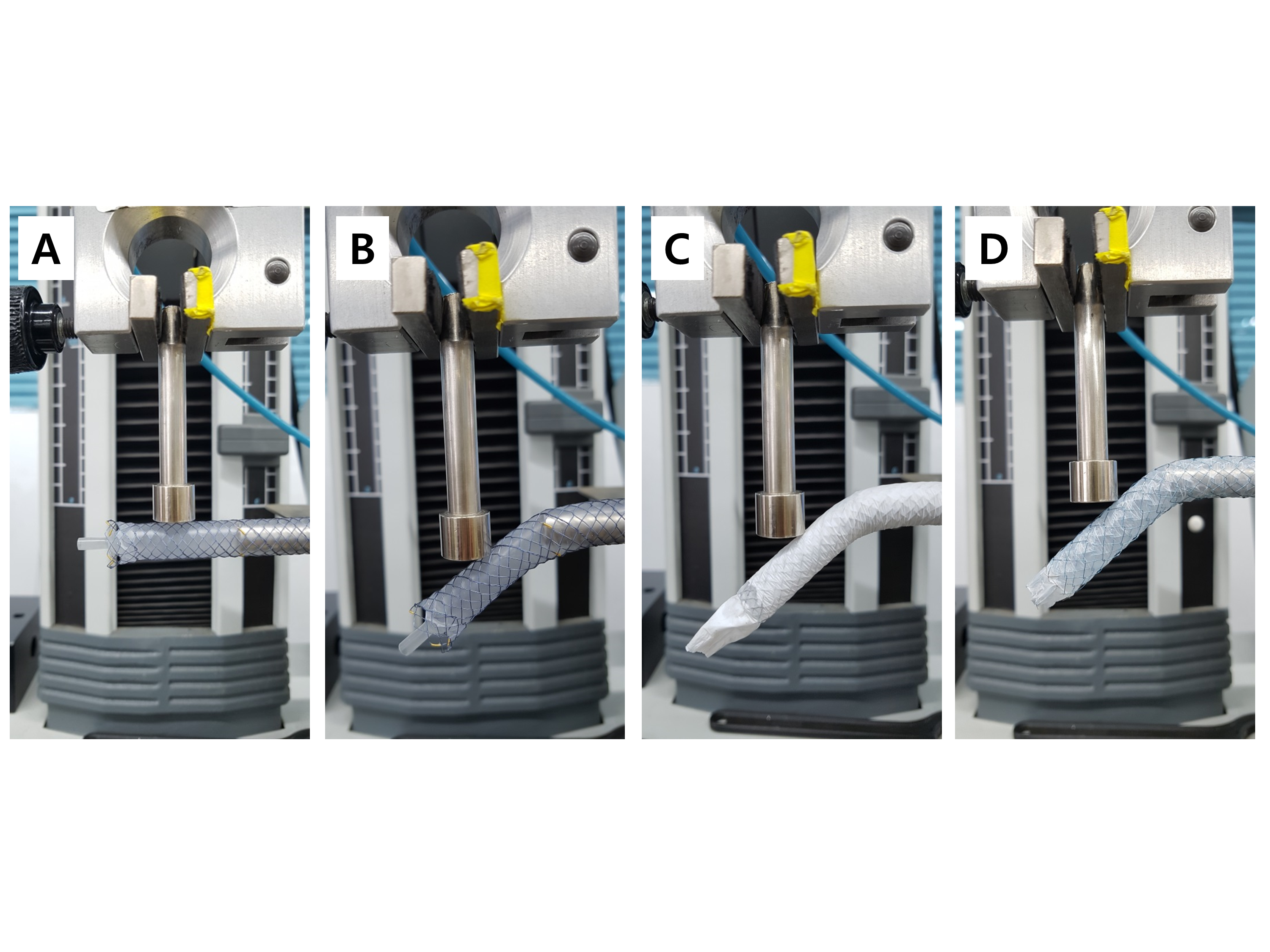

Supplement: Supplementary file 2 — Figure S2. Axial force measurement setup. Axial force was measured using the UTM (Lloyd Instruments LRX PLUS, AMETEK Inc., PA, USA). With exception of the distal end of SEMS with ARV about 40 mm, all SEMSs were fixed to the frame. Axial force was measured by bending the unfixed area. A and B. A wine glass-shaped ARV SEMS. C. A windsock type ARV SEMS. D. A funnel type ARV SEMS. (TIF 4454 kb) [file 12876_2018_878_MOESM2_ESM.tif]
